# Case Report: Primary Peritonitis as the Onset of Pediatric Ménétrier's Disease

**DOI:** 10.3389/fped.2020.589853

**Published:** 2021-01-13

**Authors:** Ana Barrés-Fernández, Andrés Piolatti-Luna, José Rafael Bretón-Martínez, Elena Crehuá-Gaudiza, Carmen Quiñones-Torrelo, Anaïs Moscardó-Navarro, Cristina Fuertes-Latasa, Cecilia Martínez-Costa

**Affiliations:** ^1^Pediatric Department, Hospital Clínico Universitario, Valencia, Spain; ^2^Department of Pediatrics, University of Valencia, Valencia, Spain; ^3^Clinic Biochemistry and Molecular Pathology Department, Hospital Clínico Universitario, Valencia, Spain; ^4^Department of Pathology, Hospital Clínico Universitario, Valencia, Spain

**Keywords:** Ménétrier's disease, protein-losing enteropathy, hypertrophic gastropathy, case report, primary peritonitis

## Abstract

**Introduction:** Primary peritonitis (PP) and Ménétrier's Disease (MD) are both rare conditions among pediatric population. Although about 150 MD cases have been described in the scientific literature to date, its onset with a PP is an unusual condition.

**Case Presentation:** We present a case of an 11-year-old boy who was admitted to our unit because of abdominal pain and distension. Complementary tests showed ascites, bilateral pleural effusion, leukocytosis, increased acute phase reactants and hypoproteinemia with hypoalbuminemia. Laparoscopy ruled out appendicitis or visceral perforations and exposed purulent peritoneal fluid, compatible with PP. Biochemical stool analysis showed increased clearance of alpha-1-antitrypsin, which was consistent with a protein-losing enteropathy. Gastroscopy findings were compatible with MD. The clinical course was favorable and he had no recurrence after 12 months of follow-up.

**Conclusion:** PP can be the first clinical manifestation of pediatric MD. Knowledge of MD and its generally benign nature in children is important in order to avoid excessive testing and unnecessary treatment.

## Introduction

Primary Peritonitis (PP) is an acute inflammatory process of the peritoneal cavity with infection of ascitic fluid and no apparent intra-abdominal source. It accounts for 1–3% of acute abdominal emergencies in children (1–4). Ménétrier's Disease (MD) is relatively common among adults, but is extremely rare in the pediatric population, with about 150 cases described in the scientific literature to date (5–7). We present an unusual case of PP as the onset of MD in a boy.

## Case Report

An 11-year-old boy was assessed at the Pediatric Emergency Room because of a 3-day history of abdominal pain and distension. He was correctly vaccinated and had no history of interest, except for a self-limited gastroenteritis, 14 days prior to the beginning of this episode.

**Figure 1:**
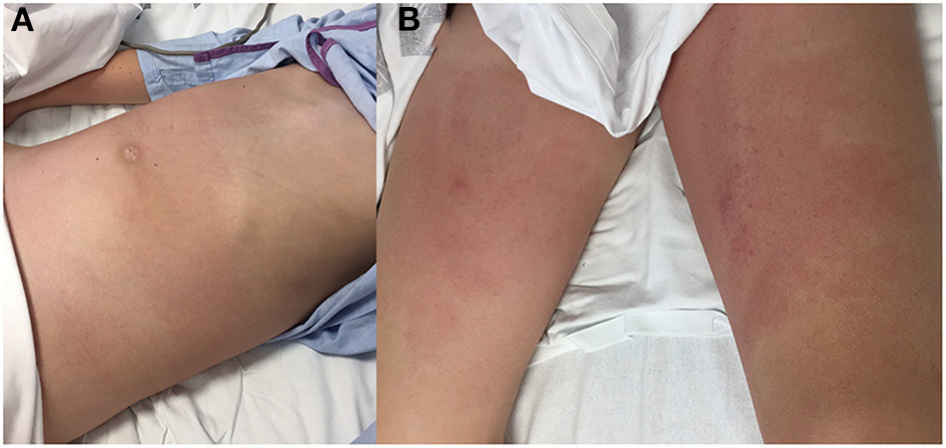
Physical examination: abdominal distension and non-pruritic erythematous plaques on the abdominal wall **(A)**, pubis and proximal segments of both lower limbs **(B)**.

The patient exhibited regular appearance due to spontaneous pain that intensified with movement, fever (38.3°C) and tachycardia (124 bpm) with normal blood pressure (104/62 mmHg). An increase of 3.5 kg over his usual weight was estimated. Physical examination revealed non-pruritic erythematous plaques on the abdomen, pubis and proximal segments of both lower limbs (Figure 1). The abdominal exam was notable for distension, diffuse pain and tenderness. The lower back was painful upon palpation. He had pitting edema at the abdominal wall, lumbar region, and distal segments of the lower limbs. Auscultation of the lungs showed decreased vesicular breath sounds at the right base.

**Figure 2:**
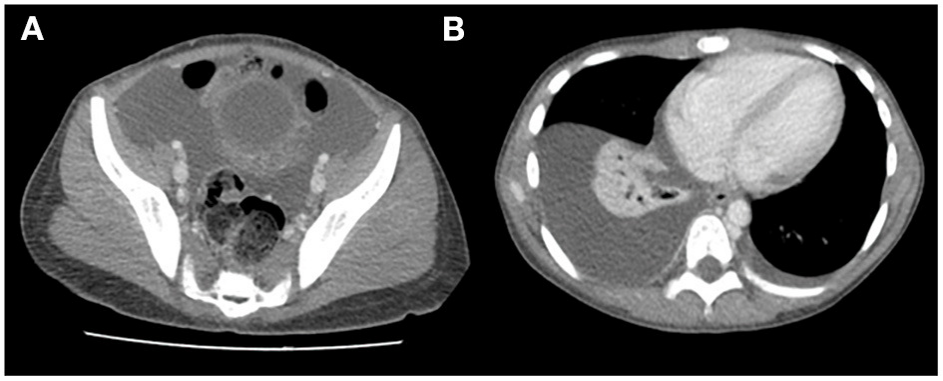
Abdominal CT: ascites **(A)**, bilateral pleural effusion **(B)**, and lumbar subcutaneous edema **(A)**.

Abdominal radiograph was performed showing a probable mass effect on loops of the transverse and descending colon without signs of obstruction. Chest radiograph revealed right pleural effusion. Abdominal CT with contrast was prioritized over ultrasound to better characterize the suspected abdominal mass. It showed ascites, bilateral pleural effusion, lumbar subcutaneous edema and right lower lobe atelectasis with normal cardiac size (Figure 2). Blood analysis revealed an increased white blood cell count of 37.48 × 10^9^/L (neutrophils 34.30 × 10^9^/L), hemoglobin and platelet count in the normal range and no abnormalities in peripheral blood smear. Increased acute phase reactants (C-Reactive Protein 345 mg/L, procalcitonin 2.77 ng/ml), hypoproteinemia with hypoalbuminemia (4.2 g of total proteins/dL with 1.9 g of albumin/dL) and hypogammaglobulinemia (IgG 137 mg/dL, IgG-1 1,060 mg/L, IgG-2 317 mg/L, IgG-3 139 mg/L, IgA 43 mg/L, other isotypes within normal ranges) were detected. Ions, amylase levels, liver enzymes, renal function parameters were in normal range. Biological parameters of heart function such as troponins and N-terminal prohormone of brain natriuretic peptide (NT-pro-BNP), were also tested as workup of ascites and pleural effusion, with normal results. The urine study did not show significant proteinuria or hematuria.

With an initial diagnosis of peritonitis and pleural effusion, hospital admission was decided and intravenous antibiotic therapy with cefotaxime was initiated. An exploratory laparoscopy was performed 24 h after admission, exposing purulent peritoneal fluid and ruling out appendicitis or visceral perforations. A routine appendectomy was performed with no abnormal anatomopathological findings. Therefore, secondary peritonitis was excluded. Samples of ascitic fluid were tested for cytobiochemical analyses (562 cells/μL, 57% polymorphonuclear; pH 7.34; glucose 31 mg/dL; protein 0.12 g/dL; LDH 32 U/mL; triglycerides 54 mg/dl) and microbiological studies (Gram and auramine stains, bacterial and mycobacterial cultures, as well as polymerase chain reaction for *Mycobacterium tuberculosis* DNA were all negative). Due to the prevalence of methicillin-resistant *Staphylococcus aureus* in our environment (~15%), vancomycin was added, and fluid restriction was prescribed with strict monitoring of fluid balance and daily weight. With the suspicion of PP with hypoproteinemia, complementary examinations dismissed nephrotic syndrome, liver failure, alpha-1-antitrypsin (α1-AT) deficiency, diabetes mellitus, lupus erythematosus, giardiasis, celiac disease, tuberculosis and immunodeficiencies. Biochemical stool analysis showed elevated α1-AT (1.9 mg/g of stool; reference value < 0.3 mg/g) and increased clearance of α1-AT (107.7 mL/24 h; reference value < 27 mL/24 h), compatible with protein-losing enteropathy (PLE).

He had a favorable clinical course with a gradual decrease in inflammatory parameters, progressive improvement in serum protein and gamma globulin levels, and total reabsorption of pleural effusion and peritoneal fluid during admission. The patient completed 21 days of antibiotic therapy (14 days of intravenous cefotaxime and 10 days of intravenous vancomycin and, after discharge, 7 days of oral amoxicillin-clavulanic acid).

**Figure 3:**
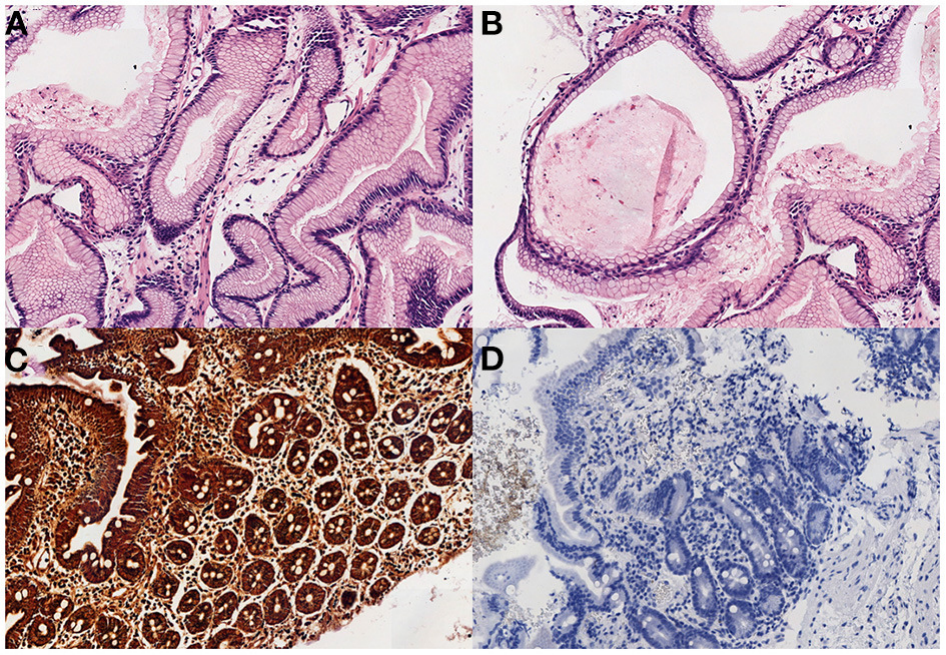
**(A)** Elongated and tortuous (corkscrew) foveolar glands, with edematous lamina propia (H-E x200). **(B)** Cystic and dilated glands, with loss of parietal cells (H-E x100). **(C,D)** The study for *H. pilory* and CMV was negative in our patient. (3. Warthin-Starry x100, 4. CMV x100).

Further studies to identify the cause of this PLE included a normal colonoscopy and a gastroscopy that showed a very erythematous and discretely edematous mucosa and enlarged gastric folds in the body and fundus of the stomach. Gastric mucosal biopsies revealed an extensive and significant hyperplasia of the foveolar mucosecretory epithelium, causing elongated and tortuous foveolar glands with loss of parietal cells, and edematous lamina propia. No signs of lymphoplasmacytic or neutrophilic infiltrate, intraepithelial lymphocytosis, pathogenic microorganisms or intracellular cytomegalovirus (CMV) inclusions were identified. Duodenal mucosa was preserved. These findings were compatible with MD. The histochemical study for *Helicobacter pylori* and the immunohistochemical study for CMV were negative (Figure 3). CMV serology was also negative.

**Figure 4:**
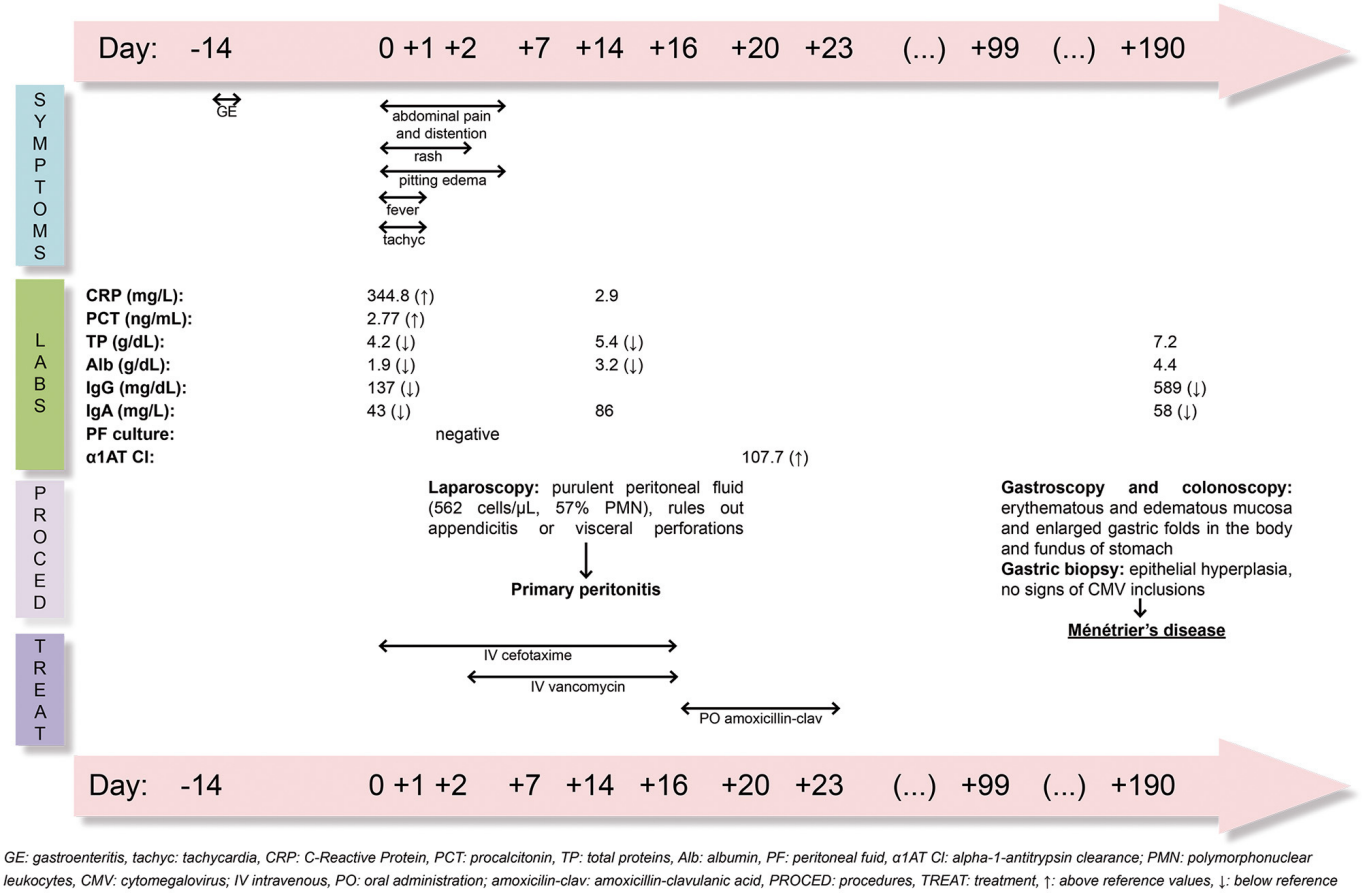
Timeline in days of clinical symptoms, laboratory findings, procedures and treatment of PP and MD.

In spite of the initial hypogammaglobulinemia of our patient, no intravenous immunoglobulins were administered to prevent the risk of new pyogenic bacterial infections since he showed a good clinical evolution and his plasmatic gammaglobulin and protein levels spontaneously increased slowly along the next weeks. Analytical controls performed 6 months later showed a complete recovery of total plasma proteins and albumin levels. Immunoglobulin levels normalized 10 months after hospital admission. After 12 months of follow-up, the patient has not presented any recurrence. A summary of his clinical course is showed in Figure 4.

## Discussion and Conclusion

Primary peritonitis, also called Spontaneous Bacterial Peritonitis (SBP), defined as bacterial peritonitis in the absence of an evident intra-abdominal focus of infection, such as intestinal perforation, is an infrequent cause of acute abdomen in the pediatric population (1, 2, 8–11). According to some pediatric series, it is more frequent in females (85–95%) and in the 4–9-year-old age group (1, 2, 4, 12). However, this case was an 11-year-old male. Although most pediatric cases occur spontaneously without underlying pathology (1, 2, 8, 12), it has been associated with diseases that cause hypoproteinemia and/or immunodeficiency such as nephrotic syndrome, liver disease, intestinal lymphoma or α1-AT deficiency (1, 9, 11–13). Patients carrying a ventriculoperitoneal shunt seem to have a higher risk to develop SBP, since spinal fluid may decrease the bacteriostatic activity of peritoneal fluid through various mechanisms (14). PP is also associated to disorders causing PLE in adults (15). Our case is an infrequent debut of MD with PP in a boy.

The most frequent clinical manifestation of PP is an acute onset of abdominal pain with or without peritonism; while fever, vomiting, and diarrhea are less frequent (1, 3, 9, 12). Periumbilical erythema is a rare sign of peritonitis (12), however, our patient initially presented erythematous plaques on the abdomen, pubis, and proximal segments of both limbs. The diagnosis is usually confirmed with laparoscopy. In our case, pancreatitis and visceral lesions were ruled out; purulent peritoneal fluid was tested with ≥250 polymorphonuclear cells/mm^3^ and ascitic fluid cultures were negative probably due to previous antibiotic treatment. All these findings, with no history of antibiotic treatment within the last 30 days, have been defined as culture-negative neutrocytic ascites (CNNA), a subset of SBP (16).

Ascitic fluid culture is negative over 50% of the samples, which may be influenced by the fact that most patients receive antibiotics prior to the diagnostic surgical procedure (4, 8), as in our case. In the pediatric age, *Streptococcus pneumoniae* is the most frequently isolated microorganism followed by Group A streptococcus (GAS) (3, 10, 12). GAS PP has been identified as the focus of 5% of GAS invasive infection in children in Finland (17) and other studies show that a possible association between GAS pharyngitis and PP in children could exist (3, 10). In the patient throat swab rapid antigen detection test (RADT) for GAS and culture were both negative. A study suggested a high sensitivity and specificity of RADT for GAS performed on deep-tissues including peritoneal fluid (18), although it was not performed in this case. Other microorganisms described are *Escherichia coli, Klebsiella pneumoniae, Enterococcus* spp., *Staphylococcus aureus* and some viruses (1, 9, 13, 19). The measurement of calprotectin and microRNA-155 in ascitic fluid can be used as biomarkers of bacterial infection of ascitic fluid (20). The detection of pneumococcal capsule antigens by immunochromatography, and polymerase chain reaction (traditional or multiplex for bacteria and viruses, as well as the amplification and sequencing of 16S rRNA) have been used for the microbiological analysis in samples with negative culture (21, 22) but these techniques were not applied in our case.

Cefotaxime is considered the antibiotic treatment of choice (1, 9, 12). In our case, due to the negative culture of the peritoneal liquid and the 15% prevalence of methicillin-resistant *Staphylococcus aureus* in our area, vancomycin was added to cefotaxime. The recommended duration of antibiotic treatment is 10–14 days for *Streptococcus* (9, 10, 19) and 10 days−3 weeks for gram-negative microorganisms (19). In the present case, as microbiological characterization was not possible, the patient received 3 weeks of antibiotic treatment. The clinical course, when detected and treated in time, is favorable in most cases (8, 12).

Ménétrier Disease is a very rare PLE in children. Since the first case of MD presented in 1888 (23), about 150 cases have been reported in the pediatric age (5–7). MD is characterized by hypoalbuminemia secondary to protein loss through the gastrointestinal mucosa, resulting in peripheral edema, ascites or even anasarca (7, 23, 24). Ascites combined with hypogammaglobulinemia results in a favorable setting for the development of PP. In many previously reported MD cases, including this one, a prodrome of abdominal pain, anorexia, and vomiting is described ~1 or 2 weeks before presentation (5, 6, 25). This is believed to be due to irritation of the gastric mucosa and death of epithelial cells during initial exposure to an unknown injurious agent (5). Most pediatric studies seem to point toward an infectious etiology, based on the presence of CMV antibodies in serum or its identification in gastric biopsy, and a self-limited course (with an average duration of 5 weeks) (5, 6, 24–26).

Increased values of α1-AT and its clearance in isolated samples of feces, point toward the diagnosis of a PLE, the latter being a more accurate method. α1-AT is an antiproteolytic plasma protein, absent in foods except for human milk and synthesized by the liver. Although it can be destroyed when the pH is lower than 3 (27), as it is the case in the stomach, it isn't degraded by intestinal proteases nor reabsorbed, so its determination in feces is a faithful marker of albumin escape to the intestinal lumen, being an excellent marker of PLE (28). Determination of α1-AT is not considered an appropriate method in breastfed infants, and its value could be falsely low if ranitidine or proton pump inhibitors (PPIs) are not used when protein leak occurs mainly though the gastric mucosa (27, 28). Our patient did not receive ranitidine or PPIs, suggesting a very high gastric protein loss or leakage through other gastrointestinal tract locations.

Definitive diagnosis of MD is obtained through imaging tests such as endoscopy, barium studies or ultrasound showing foveolar hyperplasia with glandular atrophy associated with enlarged gastric folds, plus a gastric biopsy with gastric hypertrophy and epithelial hyperplasia on microscopy, predominantly in the fundus and gastric body (5, 7, 29), as in our case. Other findings include gastric mucosa thickening with decreased main and parietal cells along with basal cystic dilatation of the gastric glands, which would facilitate protein loss at the gastrointestinal level and would subsequently lead to deep hypoalbuminemia (5, 7, 29). The changes described in the histology can be produced by some infectious agents. The most frequent is CMV (5–7, 25, 26), followed by *H. pylori* (7, 26, 30). Other less prevalent are *Mycoplasma pneumoniae* (31), *Giardia lamblia* and Herpes virus (29). However, no evidence of infection by CMV, *H. pylori* or other microorganisms was detected in our patient.

Abdominal CT usually shows transmural thickening of the stomach in the fundus and body with gastric folds abnormally thickened (7, 24). After the definitive diagnosis of MD, the CT images were reviewed carefully with the radiologists who observed some prominent gastric folds in the greater curvature of the stomach, but they also remarked that the CT wasn't performed in the ideal conditions to evaluate the typical images of MD.

In pediatric population, MD treatment is supportive and includes a high-protein diet and analgesics (5–7, 24). Some patients may require PPIs, anticholinergics steroids, diuretics (5–7, 24), intravenous immunoglobulins (30), and even albumin infusions in cases with severe and prolonged hypoalbuminemia (24, 32). Antiviral treatment with valganciclovir or ganciclovir may be considered when proved CMV infection in immunodeficient children or if the disease persists for more than 2–4 weeks (5, 6, 24, 32). In addition, cetuximab, a monoclonal antibody that blocks the epidermal growth factor receptor (EGFR) signaling, has been used for adult patients with successful results (33).

Given the favorable evolution and the frequent spontaneous resolution of the disease, the progressive recovery of protein and immunoglobulin levels in blood and the absence of new invasive infections, the administration of intravenous immunoglobulins was discarded in our case (34, 35). In the pediatric population, MD is self-limited, with a good prognosis and no tendency to recurrence. In our case, the patient did not require additional treatments and has not presented new symptoms to date. On the other hand, MD in adults is usually chronic, with a tendency to malignancy, sometimes requiring aggressive treatment such as gastrectomy (5–7, 26, 33).

In conclusion, although rarely encountered in healthy children, PP is a potentially life-threatening process that should be included in the differential diagnosis of acute abdominal pain, especially if it is associated with edema. As shown in this clinical case, PP and MD may coexist in children, although the clinical onset in this case seemed to be related to PP. Knowledge of MD and its generally benign nature in children is important in order to avoid unnecessary complementary tests or treatments.

## Data Availability Statement

The original contributions presented in the study are included in the article/Supplementary Materials, further inquiries can be directed to the corresponding author/s.

## Ethics Statement

Written informed consent was obtained from the minor's legal guardian for the publication of any potentially identifiable images or data included in this article.

## Author Contributions

JB-M, EC-G, and CM-C conceived the initial manuscript, critically reviewed, and revised the final manuscript. AB-F, AP-L, and CF-L drafted the initial manuscript, reviewed and revised the final manuscript. CQ-T and AM-N drafted the initial manuscript, implemented the biochemical and pathological procedures, respectively, and reviewed and revised manuscript. All authors provided critical feedback, approved the final version of the submitted manuscript, agreed to be accountable for all aspects of the work and ensured that questions related to the accuracy or integrity of any part of the work were appropriately investigated and resolved.

## Conflict of Interest

The authors declare that the research was conducted in the absence of any commercial or financial relationships that could be construed as a potential conflict of interest.

## Abbreviations

PP: Primary peritonitis
MD: Ménétrier's Disease
CT: Computerized tomography
NT-pro-BNP: N-terminal prohormone of brain natriuretic peptide
PLE: Protein-Losing Enteropathy
CMV: Cytomegalovirus
SBP: Spontaneous Bacterial Peritonitis
CNNA: Culture-negative neutrocytic ascites
GAS: Group A streptococcus
RADT: rapid antigen detection test
α1-AT: alpha-1-antitrypsin
PPIs: Proton pump inhibitors
EGFR: epidermal growth factor receptor.
