# Supplementary material for: Case Report: Primary Peritonitis as the Onset of Pediatric Ménétrier's Disease
Source: Front Pediatr. 2021 Jan 13;8:589853. doi: 10.3389/fped.2020.589853 (PMC7838494; doi:10.3389/fped.2020.589853)
Supplement: Supplementary file 1 [file Data_Sheet_1.PDF]

CARE case report guidelines

CARE Checklist of information to include when writing a case report

(cc) BY-NC-ND

Topic	Item	Checklist item description	Reported on Line
Title	1	The diagnosis or intervention of primary focus followed by the words "case report"	Lines 1-2
Key Words	2	2 to 5 key words that identify diagnoses or interventions in this case report, including "case report"	Lines 20-21
Abstract (no references)	3a	Introduction: What is unique about this case and what does it add to the scientific literature?	Lines 23-25
	3b	Main symptoms and/or important clinical findings	Lines 26-27
	3c	The main diagnoses, therapeutic interventions, and outcomes	Lines 27-32
	3d	Conclusion—What is the main "take-away" lesson(s) from this case?	Lines 33-35
Introduction	4	One or two paragraphs summarizing why this case is unique (**may include references**)	Lines 37-41
Patient Information	5a	De-identified patient specific information.	Lines 43-45
	5b	Primary concerns and symptoms of the patient.	Lines 46-48
	5c	Medical, family, and psycho-social history including relevant genetic information	Not applicable
	5d	Relevant past interventions with outcomes	Lines 44-45
Clinical Findings	6	Describe significant physical examination (PE) and important clinical findings.	Lines 46-52
Timeline	7	Historical and current information from this episode of care organized as a timeline	Lines 333 -334. Figure 4
Diagnostic Assessment	8a	Diagnostic testing (such as PE, laboratory testing, imaging, surveys).	Lines 46-66; 68-83; 88-97
	8b	Diagnostic challenges (such as access to testing, financial, or cultural)	Not applicable
	8c	Diagnosis (including other diagnoses considered)	Lines 55-56;64-66;68-71;77-82
	8d	Prognosis (such as staging in oncology) where applicable	Lines 154-157
Therapeutic Intervention	9a	Types of therapeutic intervention (such as pharmacologic, surgical, preventive, self-care)	Lines 67-71; 75-77; 83-87
	9b	Administration of therapeutic intervention (such as dosage, strength, duration)	Lines 85-87
	9c	Changes in therapeutic intervention (with rationale)	Lines 75-76
Follow-up and Outcomes	10a	Clinician and patient-assessed outcomes (if available)	Lines 98-103
	10b	Important follow-up diagnostic and other test results	Lines 83-85; 98-103
	10c	Intervention adherence and tolerability (How was this assessed?)	Lines 83-85; 98-103
	10d	Adverse and unanticipated events	There wasn't any adverse and unanticipated events.
Discussion	11a	A scientific discussion of the strengths AND limitations associated with this case report	Line 197-201
	11b	Discussion of the relevant medical literature **with references**.	Lines 105-201
	11c	The scientific rationale for any conclusions (including assessment of possible causes)	Lines 150-151; 197-201
	11d	The primary "take-away" lessons of this case report (without references) in a one paragraph conclusion	Lines 197-201
Patient Perspective	12	The patient should share their perspective in one to two paragraphs on the treatment(s) they received	Patient and family were satisfied with treatment. In this case we consider this information is not necessary.
Informed Consent	13	Did the patient give informed consent? Please provide if requested	Yes ☑ No ☐
